# Wee1 Kinase: A Potential Target to Overcome Tumor Resistance to Therapy

**DOI:** 10.3390/ijms221910689

**Published:** 2021-10-01

**Authors:** Francesca Esposito, Raffaella Giuffrida, Gabriele Raciti, Caterina Puglisi, Stefano Forte

**Affiliations:** IOM Ricerca srl, Viagrande, I-95029 Catania, Italy; francesca.esposito@grupposamed.com (F.E.); raffaella.giuffrida@grupposamed.com (R.G.); gabriele.raciti@grupposamed.com (G.R.); caterina.puglisi@grupposamed.com (C.P.)

**Keywords:** Wee1 kinase, cell cycle, tumor resistance

## Abstract

During the cell cycle, DNA suffers several lesions that need to be repaired prior to entry into mitosis to preserve genome integrity in daughter cells. Toward this aim, cells have developed complex enzymatic machinery, the so-called DNA damage response (DDR), which is able to repair DNA, temporarily stopping the cell cycle to provide more time to repair, or if the damage is too severe, inducing apoptosis. This DDR mechanism is considered the main source of resistance to DNA-damaging therapeutic treatments in oncology. Recently, cancer stem cells (CSCs), which are a small subset of tumor cells, were identified as tumor-initiating cells. CSCs possess self-renewal potential and persistent tumorigenic capacity, allowing for tumor re-growth and relapse. Compared with cancer cells, CSCs are more resistant to therapeutic treatments. Wee1 is the principal gatekeeper for both G2/M and S-phase checkpoints, where it plays a key role in cell cycle regulation and DNA damage repair. From this perspective, Wee1 inhibition might increase the effectiveness of DNA-damaging treatments, such as radiotherapy, forcing tumor cells and CSCs to enter into mitosis, even with damaged DNA, leading to mitotic catastrophe and subsequent cell death.

## 1. Introduction

The cell cycle is a finely regulated process, where a series of growth and development steps alternate and several molecules are involved as negative or positive regulators. Proteins belonging to the highly evolutionarily conserved family, known as cyclin-dependent kinases (Cdks), interact with specific partner proteins named cyclins. The variation in cyclin protein levels is the mechanism through which cells progress through the cell cycle, alternating the phases that follow one another in a specific temporal sequence [1]. Besides the kinases, even their molecular counterparts, the phosphatases, take part in the cell cycle. Therefore, kinases and phosphatases alternatively regulate the same targets to allow for the correct conclusion of the process and to dynamically respond to the events.

The major events of the cell cycle are the S-phase, where the DNA replication occurs, and the M-phase, which finally leads to the generation of the two daughter cells. These phases are connected through “gap” steps (G1 and G2 respectively), during which, the cell prepares itself through modifications of its transcriptional activity to synthetize the molecules that are required for the following event.

Among the various steps, there are mechanisms of control, known as checkpoints, which temporarily stop the process when mistakes occur to avoid the transmission of mistakes that would compromise the result [2].

In normal cells, DNA damage is usually repaired via G1 phase arrest. In tumor cells, G1 checkpoint deficiencies can occur, especially in p53-deregulated cells. In these cells, the G2 checkpoint has the crucial role of repairing endogenous and exogenous DNA damage [3,4]. Consequently, targeting the effectors involved in the G2 checkpoint is a promising strategy for cancer therapy [5,6].

Wee1 belongs to a family of protein kinases that activate the G2/M checkpoint of the cell cycle in response to double-stranded DNA breaks (DDB) [7,8]. It is involved in the terminal phosphorylation and inactivation of Cdk1/Cdc2-bound cyclin B on its Tyr15 residue, resulting in cell cycle arrest at G2 and, therefore, a delayed entry into mitosis after DNA damage [9,10].

## 2. Wee1 Family

Wee1 is one of the most important gatekeepers for both the G2/M checkpoint and S-phase. While its role in regulating the entry into mitosis is well known since its discovery, its involvement in the S-phase was discovered only recently. Since DNA synthesis and mitosis are tightly connected, when replication errors occur, the regulatory mechanism can slow down or temporarily stop the S-phase. This allows for DNA repair before the onset of mitosis, which will fix genetic mutations and/or chromosomal aberrations in the genome of daughter cells.

Wee1 belongs to a family of protein kinases, consisting of three members in humans, including PKMYT1 (membrane-associated tyrosine- and threonine-specific cdc2-inhibitory kinase) and two Wee1 kinases (Wee1 and Wee1B). They show sequence similarity on their kinase domain but differ regarding their localization, temporal expression and regulation. 

**Figure 1:**
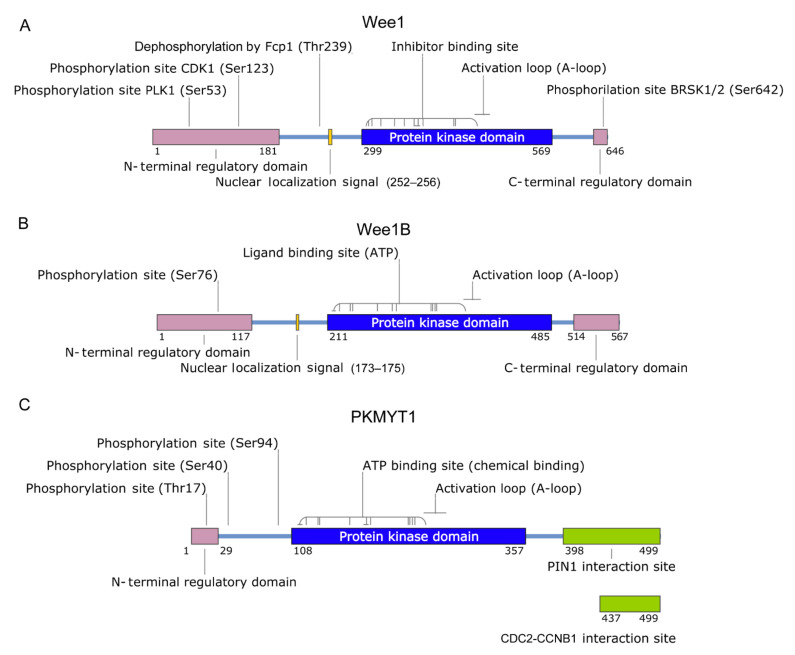
Schematic representation of Wee1 (**A**), Wee1B (**B**) and PKMYT1 (**C**) domain structures, interaction and post-translational modifications sites.

Human Wee1, also known as Wee1A or Wee1Hu, is a kinase of 646 amino acids with a molecular weight of 94 kDa comprising three domains: an N-terminal regulatory domain, a central kinase domain and a C-terminal regulatory domain [9,11,12]. The N-terminal domain is the longest and contains two phosphorylation sites (S53 and S123), which are involved in protein degradation through the proteasome. In fact, this domain presents three PEST (Pro-Glu-Ser-Thr) regions, which are found in many eukaryotic proteins and are characterized by a rapid turnover. A nuclear localization signal, which is in the same domain, is responsible for its subcellular localization (Figure 1).

Even if Wee1 acts as a tyrosine kinase, its catalytic domain is similar to the ones found on serine/threonine kinases rather than those found on tyrosine kinases. In fact, Wee1 acts on the Y15 residue on Cdk1-cyclin B. Its structural features suggest that Wee1 may have evolved from a serine/threonine kinase through mutations that somehow might have directed it to acquire the ability to phosphorylate tyrosine residues [12]. Finally, the C-terminal regulatory domain is very short and presents an S642, which constitutes a binding site for the chaperone 14-3-3.

Wee1B was identified for the first time in Xenopus oocytes. It shares with Wee1 its predominant nuclear localization and the ability to inactivate Cdk1-cyclin B through phosphorylation. However, higher levels of its mRNA were observed in mature oocytes with a marked decrease after fertilization, while Wee1 is more expressed in the zygotes. This suggests a different action during the early phase of embryogenesis, with Wee1B being more involved in the early steps and Wee1 in the latter steps [8].

While Wee1 and Wee1B are prevalently nuclear, PKMYT1 is not. Its C-terminal domain is anchored at the endoplasmic reticulum and Golgi apparatus; in addition, it binds Cdk1-cyclin B. Furthermore, it is a dual-specificity kinase and phosphorylates Cdk1-cyclin B in both Y15 and the adjacent threonine 14 [13,14]. PKMYT1 prevents cell entry in the M-phase in two ways: it maintains Cdk1-cyclin B inactivity and, at the same time, prevents the translocation of the complexes into the nucleus [15]. Moreover, it exhibits more restricted substrate specificity, acting on Cdk1 but only partially on Cdk2 [16].

The Wee1 gene, which is localized in the distal short arm of the human chromosome 11 [17], was first discovered in the fission yeast Schizosaccharomyces pombe. It is involved in the controlled onset of mitosis once the cell reaches the right size [18]. Moreover, a gene dosage effect was observed: several experiments on yeast indicated that Wee1 could delay progression through G2 into mitosis and this effect was more evident when its expression increased [9,19]. Orthologues of the yeast Wee1 gene were identified not only in human cells [20,21] but in several other eukaryotes, such as mouse, chicken, lizard, zebrafish and the African clawed frog [22,23,24,25,26,27,28]. In addition to the above-mentioned members of the Wee1 family, other paralog genes have been identified. They comprise four eukaryotic translation initiation factor genes (EIF2AK1, EIF2AK2, EIF2AK3, EIF2AK4), the serine/threonine kinase 35 (STK35) and the PDLIM1 interacting kinase 1 like (PDLIM1) genes.

Its activity is sustained by various molecules to acquire more stability, a prolonged half-life and, consequently, increased biochemical activity. In particular 14-3-3 proteins, which are molecular chaperones that interact with many proteins that are involved in signal transduction, are likely the most important [29]. Their binding during the interphase induces a conformational change at the N-terminal domain of Wee1, hiding its site for degradation. Consequently, Wee1 becomes more stable and thus enhances kinase activity. During mitosis, 14-3-3 instead loses its binding with Wee1, allowing for an easier inactivation [30,31,32].

Moreover, Hsp90 and MIG6 support Wee1 stability. Heat shock protein 90 (Hsp90), which is one of the most abundant and evolutionarily conserved chaperones, interacts with Wee1 and maintains it in its native conformation [32,33,34,35]. MIG6 is a known EGFR inhibitor whose expression is upregulated with cell growth. Sasaki and his collaborators recently identified a new EGFR-independent role in cell cycle progression. They demonstrated that MIG6 stabilizes Wee1, hindering the recruitment of β-TrCP-SCF and the subsequent proteasomal degradation [36].

## 3. Wee1 in Cell Cycle Events

Wee1 is predominantly localized in the nucleus, where it coordinates and ensures the proper DNA replication and, at the same time, prevents premature mitosis [37,38].

Wee1 negatively regulates the G2/M transition that acts on the Cdk1-cyclin B complex (also known as mitosis promoting factor (MPF)), which, once activated, triggers all events leading to the onset of mitosis [39,40,41]. Wee1 phosphorylates Cdk1 (also known as cell division cycle 2 (Cdc2)) on the Y15 residue [21,42] within its ATP-binding site in the catalytic subunit, while PKMYT1 phosphorylates it on both the Y15 and T14 residues [13,14,16]. This phosphorylation inactivates Cdk1 during the interphase. Its activity is contrasted with Cdc25 phosphatases, whose levels change in a specular way compared to those of Wee1. Cdc25 dephosphorylates Y15 and T14 and, together with higher cyclin B levels, which promote the phosphorylation on its T161 residue by Cdk-activating kinase (CAK), activates Cdk1 [43,44].

**Figure 2:**
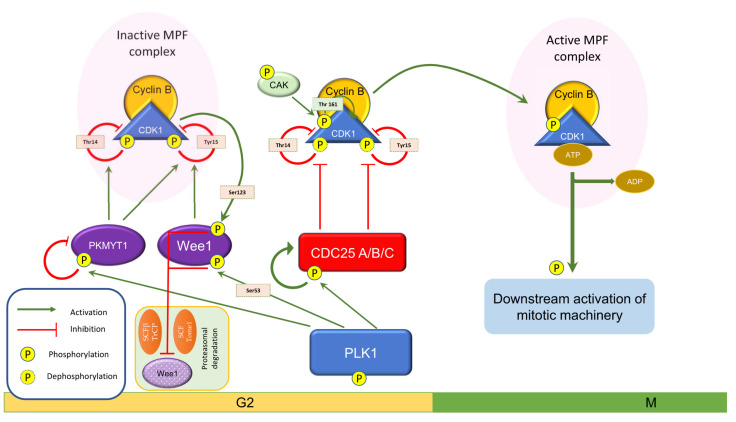
Schematic representation of the components involved in G2/M cell cycle transition.

The downregulation of Wee1 promotes entry into mitosis. This is usually achieved via both decreased synthesis and proteolytic degradation [38]. When Wee1 is phosphorylated by Plk1 (polo-like kinase 1) and Cdk1 in S53 and S123, respectively, it becomes a target of the β-TrCP F-box protein-containing SCF E3 ubiquitin ligase (SCFβ-TrCP) or Tome-1 F-box protein-containing SCF E3 ubiquitin ligase (SCFTome-1) complexes. Both β-TrCP and Tome-1 directly contribute to Wee1 inactivation [45,46,47]. Therefore, while phosphorylation does not directly inactivate Wee1, this event induces its proteasome-dependent degradation (Figure 2).

It is important to highlight that Wee1 inactivation is enhanced by Cdk1-cyclin B itself, which triggers a feedback loop: when Wee1 levels decrease, the balance between the tyrosine kinase and Cdk1-cyclin B complexes shifts toward the latter. These, in turn, phosphorylate Wee1, promoting its recognition for proteolytic degradation. Thus, the process ensures a rapid activation of Cdk1-cyclin B to allow for a quick transition to mitosis [11,46].

During the S-phase, Wee1 acts on Cdk2 to stabilize the replication machinery, preventing unscheduled replication origins and the resulting insurgence of abnormal structures that might cause genomic instability [48]. The exact molecular mechanism is unclear: it could downregulate both Cdk2 and the Mus81-Eme1 endonuclease or Cdk1 and, only indirectly, Mus81-Eme1 activity [49,50]. In the presence of stalled forks, DSBs are induced by Mus81-Eme1 complexes to initiate recombination-mediated replication fork recovery. When downregulation that is caused by Wee1 is turned off, Cdk2 becomes hyperphosphorylated and thereby activates the Mus81-Eme1 endonuclease. Moreover, the phosphorylated form of H2A histone, namely, γ-H2AX, increases the formation foci that recruit molecules that are responsible for the DNA repair pathway. As such, the endonuclease binds to DNA, even in the absence of damage, and generates DSBs, cleaving the DNA in an unscheduled manner, thus reducing replication fork speed [49,51]. Therefore, Wee1 activity stabilizes those replication forks, which are temporarily stalled without becoming a signal to activate the DNA damage response.

Furthermore, Wee1 prevents the multiple and unscheduled activation of replication forks, leading, in turn, to the rapid consumption of the deoxynucleotide triphosphates (dNTPs) pool. Therefore, more replication forks become stalled and S-phase arrest ensues [52,53].

Recently, Vassilopoulos et al. found a novel interaction between Wee1 and anaphase-promoting complex/cyclosome (APC/C). Activated APC/C is an E3 ubiquitin ligase that targets key proteins, including cyclin B and securin, causing them to degrade, thus promoting anaphase initiation and segregation of sister chromatids. In the presence of DNA damage in the S-phase, Wee1 phosphorylates APC/C, which, as a result, becomes inactive. If Wee1 is inactivated, APC/C recruits polo-like kinase 1 (Plk-1), which degrades Wee1, and the cell cycle proceeds independently due to the integrity of the genetic information [54].

Finally, Wee1 can act as an epigenetic modifier: in the late S-phase, when DNA synthesis is completed, it phosphorylates H2B histone at the Y37 residue, suppressing the transcription of Hist1, which is the major histone gene cluster. Thus, by modifying the chromatin structure, histone overproduction is avoided. This is an evolutionarily conserved process in the eukaryotic cells, as it is observed in yeast and mammals [55].

To better understand the role of Wee1 in mammalian tissue, in 2006, the group of Prof. Deng produced *Wee1* knockout mice via gene targeting, which resulted in embryonic lethality at the blastocyst stage [56], which explained the lack of in vivo models. In 2015, the same group established conditional and tissue-specific *Wee1* mutant mice and cells. Alongside the well-known role of Wee1 in preventing premature mitotic entry, *Wee1* mutant cells revealed impaired mitosis that was characterized by delayed progression and completion without normal cytokinesis. Moreover, they found a role of Wee1 in modulating the APC/C complex, i.e., it is responsible for the degradation of mitotic regulator protein by a proteasome. Thus, *Wee1* deficiency causes increased activity of APC/C, which allows for mutant cells to progress through mitosis at the expense of genomic integrity. Finally, in the animal model with a conditional expression of Wee1 in the mammary gland, they demonstrated that Wee1 is indispensable for maintaining genomic stability and it acts as a haploid tumor suppressor since a mutant mammary gland develops tumors. In conclusion, Wee1 coordinates distinct cell-division events to permit correct segregation of genetic information into daughter cells and preservation of genomic integrity [54].

## 4. Role of Wee1 Kinase in Cancer Progression and Therapy

The expression of Wee1 was investigated in few studies, including solid tumors and hematological malignancies. Wee1 downregulation in cancer tissues was observed in most of them, suggesting a tumor suppressor role for Wee1. In stromal breast cancer, Wee1 expression was investigated using immunohistochemistry. The lower Wee1 expression in malignant tumors compared to benign ones suggests that it acts as a tumor suppressor [57]. Similarly, a cDNA array performed on colon carcinoma cell lines and human tissues showed low Wee1 mRNA expression, leading to the speculation that genetic lesions targeting cell cycle regulation occur not only at the G1-S but also the G2-M transition checkpoint during tumor development [58]. Since the Wee1-mediated inhibition of G2-M transition is an essential step after DNA damage, permitting DNA repair prior to entry into mitosis [37], the decreased expression or loss of the Wee1 gene in colon carcinoma cells suggests its potential role as a tumor suppressor [58]. The association between Wee1 downmodulation and tumorigenesis was also found in non-small-cell lung cancer (NSCLC) [59]. In this work, the authors found a correlation between low Wee1 levels and a poor prognosis, as impaired Wee1 expression provided an advantage to neoplastic cells since it caused a faster progression through the cell cycle [59]. In their work, Yoshida et al. investigated the clinicopathological and prognostic significance of cyclin B and Wee1 expressions in 79 patients affected by NSCLC. It was found that in almost two-thirds of patients analyzed, Wee1 was not expressed and the patients had a poorer prognosis and a higher recurrence rate. Moreover, their tumors tended to have a higher Ki index and PCNA-LI values, which are typical parameters of cell proliferation and malignancy potential in NSCLC. Taken together, these data suggested that a loss of Wee1 expression may both promote tumor aggressiveness and be a useful prognostic indicator.

Few studies have evaluated the role of microRNAs (miRNAs), long non-coding RNAs (lncRNAs) and small-interference RNAs (siRNAs) that are able to modulate Wee1 expression in tumor cells and their involvement in tumor progression [60,61,62,63,64]. Aside from the evidence of an inverse correlation between Wee1 protein expression and the aggressiveness of melanoma cells, Bhattacharya et al. found a link with miR-195. They demonstrated that miR-195 has an inhibitory activity on Wee1 expression in melanoma metastases [61]. Data that was obtained by assessing cell cycle analyses during simultaneous Wee1 silencing (by siRNA or miR-195) and genotoxic agents’ exposure suggested that when chemotherapy-mediated DNA damage occurs, miR-195 is able to significantly contrast the G2/M cell cycle arrest, downmodulating Wee1 in melanoma cells. [61]. In contrast, the upregulation of miR-101-3p via lncRNA NEAT1_2 in hepatocellular carcinoma cells decreased both the mRNA and protein levels of Wee1, inducing tumor radio-sensitization [60]. Comparable results were obtained in myeloid leukemia cell lines, whose Wee1 siRNA silencing resulted in a strong sensitization to cytarabine (Ara-C) [65]. While these data indicate that Wee1 acts as a tumor suppressor, several studies highlighted an increased expression of Wee1 in several types of cancer. This indicates that Wee1 may be important for cancer cells’ viability under specific circumstances. A cell viability assessment with a kinase siRNA library in different cancer cell lines demonstrated that Wee1 gene expression correlated with the Wee1 gene copy number, potentially identifying a cause of increased expression [66]. Moreover, this study demonstrated that tumor cell lines that overexpress Wee1 are more sensitive to Wee1 inhibition by siRNA, leading to abrogation of the G2/M checkpoint and consequent tumor cell death via apoptosis. In particular, thanks to this approach, authors could identify a breast cancer patient subset (luminal breast cancer) that overexpressed Wee1, where its inhibition could be suggested as a potential therapeutic strategy [66]. Interestingly, by performing a stringent in silico analysis on data obtained comparing normal versus cancer tissue, Mir et al. found increased expression of Wee1 in most cancer types (27 samples in a 35-sample data set) [67]. In their dataset, the highest Wee1 mRNA expression was measured in glioblastoma, followed by non-small-cell lung carcinoma, (non-)seminoma and colon carcinoma, whereas the other cancer types mostly showed moderate overexpression as compared to the relevant non-neoplastic control tissue [67].

The activity of Wee1 was found to be increased in patients with advanced hepatocellular carcinoma when compared with noncancerous liver tissue [68]. Moreover, Wee1 was found to be overexpressed and functionally important in medulloblastoma [69], and a high expression of Wee1 was described in glioma [70,71]. Slipicevic et al. reported an expression of Wee1 in ovarian serous carcinoma effusions. Moreover, they observed a notable increase in Wee1 levels after exposure to chemotherapy, suggesting a role for this kinase in mediating the progression of the disease. Thus, increased Wee1 expression may represent an adaptive response to the chemotherapy that allows tumor cells to repair DNA damage and thereby survive [72].

Although Wee1 expression was found to increase in vulvar squamous cell carcinomas compared to normal tissue, Magnussen et al. did not observe any significant association between disease-specific survival and Wee1 expression in patients with vulvar carcinomas [73]. These findings did not directly support the tumor suppressor role of Wee1. Moreover, Wee1 silencing by siRNA did not translate to any major alteration in viability [73]. However, in their previous study, Magnussen and colleagues linked the expression of Wee1 with the activation of cellular pathways that are crucial for the specific disease [74]. In nasopharyngeal (NP) carcinoma cell lines, Wee1 was found to be overexpressed and, consequently, cells were found to be more sensitive to its inhibition compared to NP epithelial cells, although such inhibition was not very effective in sensitizing cells to radiotherapy [75]. In melanoma cells, Wee1 overexpression showed a strong, positive correlation with markers of proliferation: cyclin A, Ki67 and cyclin D3 [74]. Wee1 silencing caused an increase in phospho p38 protein levels, indicating a role in the regulation of p38/MAPK pathway activation during p53-independent DNA damage responses [74].

Aside from the reported use of siRNA for the inhibition of Wee1 expression in different cancer models, several pharmacological inhibitors were developed and validated, both as single agents or in combination with DNA damaging agents (chemotherapy/radiotherapy) [69,76,77,78,79]. Wee1 kinase inhibition causes a significant reduction in phospho- CDK1 (Tyr15), thus promoting the accumulation of the active CDK1-cyclin B1 complex and driving premature mitotic entry. Uncontrolled and deregulated mitosis is associated with a progressive DNA damage accumulation, culminating in cell death through a mechanism that is generally known as a mitotic catastrophe. The efficacy of Wee1 inhibitors as monotherapy was confirmed by a decrease in cell viability of ovarian cancer and sarcoma cell lines [80,81].

A large number of studies demonstrated that the cellular rate of response to treatment with Wee1 inhibitors or mimics was strictly dependent on a concomitant (i) presence of TP53 mutations [82,83,84] and/or (ii) administration of DNA-damaging agents (chemotherapy including doxorubicin, cytarabine, methotrexate, cisplatin, clofarabine, etoposide, 5-fluorouracil and radiotherapy) [85,86]. Moreover, some data suggest that cells with dysfunctional p53 are more sensitive to Wee1 inhibition combined with conventional chemotherapy than those with functional p53. A possible explanation for this is that the dysfunctional G1/S DNA damage checkpoint yields TP53-mutated cells that are more dependent on stopping in G2 to repair DNA damage before entering mitosis.

While some studies described ionizing radiation (IR) [87,88] and chemotherapy [89] sensitization mediated by Wee1 inhibition to be dependent on TP53 activity, the pharmacological inhibition of Wee1, in combination with cytarabine, was shown to be effective in AML cell lines with functional p53 [90]. Van Linden demonstrated that the functionality of p53 does not influence the sensitization to antimetabolite chemotherapeutics by Wee1 inhibitors in AML cells and lung cancer cells, suggesting that the use of p53 mutation as a predictive biomarker for response to Wee1 inhibition may be restricted to certain cancers and/or chemotherapeutics, as well as the preclinical data supporting the combination [91].

Table 1 summarizes the above reported evidence observed in different cancer types. 

## 5. Radiotherapy and DNA Damage Response (DDR)

Radiation therapy is one of the crucial cancer treatment options along with surgery and chemotherapy [92]. The curative potential of radiotherapy depends on the amount of non-repairable DNA lesions that occurred in the exposed tumor tissue, thereby removing cancer cells from the clonogenic pool [93,94,95,96].

DNA integrity can be compromised by several types of exogenous and/or endogenous injuries. In particular, exposure to ionizing radiation is responsible for base and sugar damage, cross-links and both single- and double-strand breaks (SSBs and DSBs, respectively). Among these, DNA double-strand breaks (DSBs) principally contribute to radiation-induced cell death. However, cells can avoid this fate through DNA repair mechanisms. When DNA damage occurs, cells can arrest cycle progression to allow for DNA repair before cell division, which makes DNA alteration permanent. In fact, the accumulation of DNA lesions may lead to cell death and/or senescence if the damage is too severe to be repaired [97].

DNA repair mechanisms are the key determinant of tumor cell sensitivity (or resistance) to radiation and, for this reason, have gained a lot of interest in the oncology field. If the DNA repair capacity of tumor cells is not able to mitigate the severity of radiation-induced DSBs, this might result in the perpetuation of DNA damage, leading to irreparable genetic lesions that culminate in cell death. In contrast, if cells can repair radiation-induced damage, cancer cells continue to proliferate and tumors may recur [98].

In the presence of genotoxic stress, three different DNA damage repair (DDR) pathways are activated. Three phosphatidyl inositol 3-kinase-related protein kinases are the upstream molecules involved, though activated by different injury types. Ataxia telangiectasia mutated (ATM) responds to DNA-damage agents, such as IR, causing DSBs. Ataxia telangiectasia and Rad3-related (ATR) kinase detect alterations of replications (stalled replication forks and branched structures formation) that occur naturally or after ultraviolet light (UV) exposure. Like ATM, the DNA-dependent protein kinase (DNA-PK) signaling pathway is activated by DSBs under different cellular conditions, including IR exposure, environmental carcinogens and chemotherapeutic agents, or in cells with shortened telomeres.

ATM is recruited to DSBs by the Mre11–Rad50–Nbs1 (MRN) complex, whereas DNA-PK is recruited by the Ku70/Ku80 heterodimer [99]. ATR is recruited by the ATR-interacting protein (ATRIP) to replication protein A (RPA)-coated single-stranded DNA (ssDNA), which forms at stalled DNA replication forks or is generated by processing of the initial DNA damage [100,101,102]. Both ATM and ATR are able to induce chromatin modification in the presence of DNA damage through phosphorylation of H2AX, forming foci at the break sites. H2AX, once phosphorylated (creating γ-H2AX), allows for the recruitment of other proteins that take part in the repair mechanism [103]. ATM and ATR regulate the Werner syndrome protein (WRN), which is implicated in the recovery of stalled replication forks, to limit fork collapse [104] and act on BRCA-1, which serves as a scaffold to facilitate ATM and ATR to activate downstream substrates [105].

When recruited, ATM and ATR phosphorylate several substrates. Their principal downstream effectors are two kinases, namely, Chk2 and Chk1, which spread the signal to other molecules [106,107].

Chk2 acts on protein p53, which is the principal gatekeeper of the G1-phase, determining the arrest of the cell cycle. ATM can also directly regulate p53 stability, weakening its interaction with its negative regulator, namely, the MDM2 oncoprotein, whose gene is, in turn, activated by p53 itself [108].

**Figure 3:**
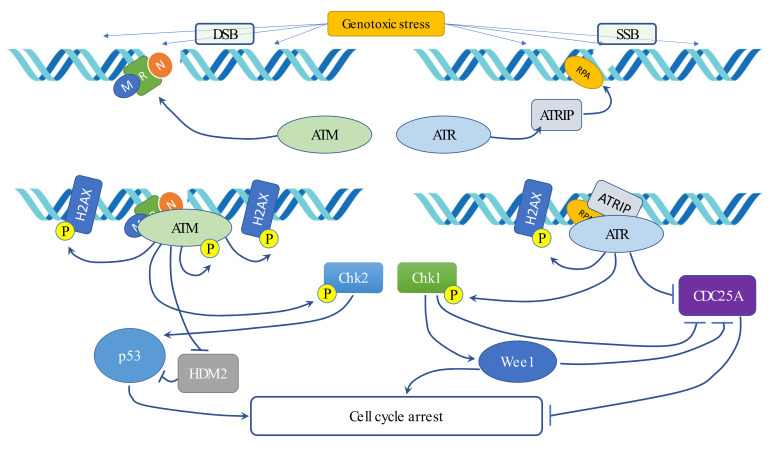
Schematic representation of the components involved in the DNA damage response.

Among Chk1 substrates, in addition to Wee1, whose function as the gatekeeper in G2/M checkpoint and S-phase was discussed above, there are the phosphatases Cdc25 (A, B and C). Their activities are directly inhibited, promoting their degradation and causing cell cycle delay not only in the G2/M checkpoint but even in other steps, providing time for DNA repair [109,110]. However, Chk1 regulates Cdc25 levels both in the presence of DNA damage and in physiological conditions, supporting its rapid turnover and avoiding an unscheduled massive DNA synthesis [111,112] (Figure 3).

Furthermore, Chk1 is required for homologous recombination repair (HRR), which is a mechanism that is essential in mammals for restoring DNA integrity after DSBs, as well as other lesions; in fact, it promotes the association of chromatin and phosphorylation of RAD51, which is a key protein in HRR [113].

Moreover, Chk1 is required for mitotic spindle checkpoint functioning. During the metaphase, chromosomes are hooked in both their sides by microtubules, forming a mitotic spindle coming from opposite poles of the cell; then, they are aligned in the central portion of the mitotic spindle and equally divided to the daughter cells, which receive a chromosomal copy each. If something at the level of alignment does not occur regularly and defects emerge, Chk1 is phosphorylated and, in turn, phosphorylates Aurora B protein kinase. This regulates BubR1 protein, which, together with Mad1 and Mad2, forms an inhibitory ternary complex with the E3 ligase APC and its activator Cdc20 in the mitotic spindle checkpoint. Thus, BubR1 is recruited at the kinetochore level and delays the passage from the metaphase to the anaphase, protecting against chromosomal segregation defects [114,115,116]. Following the DSB, DNA-PK-mediated DNA repair occurs via the non-homologous end joining (NHEJ) process. In such a process, each broken DNA end is first bound by one Ku70/80 heterodimer and two heterodimers interact together to bridge matching ends. Ku plays a crucial role in NHEJ, recruiting multiple downstream proteins, including the DNA-dependent protein kinase catalytic subunit (DNA-PKcs). In turn, DNA-PKcs interacts with Artemis and DNA ligase IV, enabling NHEJ [99,117]

## 6. Cancer Stem Cells and WEE1 Involvement in Radiation Oncology

Cancer cells are heterogeneous regarding their tumor-initiating properties. In particular, cancer stem cells (CSCs) are a small subset of a tumor population, normally representing 0.1–10% of all tumor cells, which were identified as tumor-initiating cells. CSCs possess self-renewal potential and persistent tumorigenic capacity that make them different from other tumor cells.

Experimental evidence on tumor cell transplantation, as well as in both isogenic murine models and human xenograft tumor models, demonstrated that the number of CSCs in these experimental tumors defines the therapeutic potential of radiotherapy, thus a higher proportion of CSCs correlates with a higher radio-resistance in the same histopathological tumor type [93,94,118,119,120]. Additionally, the intrinsic radiosensitivity of CSCs varies between tumors, thereby affecting their radio-curability [121,122]. A recent CSC model, which was developed by our research group, allowed for predicting the radiotherapy treatment efficacy based on the evident concordance between in vitro and in vivo CSC sensitivity to radiotherapy [121]. Notably, the specific CSC in vitro and in vivo sensitivity values correspond to patients’ responses to radiotherapy. This approach may be useful for driving clinical decisions for correct therapeutic option management [121].

As a key source of resistance to DNA-damaging treatment in oncology, CSCs were reported to improve DNA repair capacity by enhancing the activation of the DNA damage response compared with differentiated cells [123,124]. CSCs contribute to radio- and chemo-resistance through a circular mechanism: repeated cycles of DNA-damaging treatments progressively kill the non-stem cells, causing an increase in the CSC fraction within the tumor cell population. The tumor microenvironment can also influence the tumor cell sensitivity or resistance to IR. In particular, it was demonstrated that CSCs are protected from the effects of IR by hypoxia that is mediated by specific hypoxic niches [93,125]. Hypoxia might affect CSC proliferation and viability via hypoxia-inducible factors (HIFs) that can induce the expression of OCT4, MYC and NOTCH1, which are crucial for stem cell maintenance in different tissues [126,127,128]. In addition, the cell–stroma interactions, by means of the integrin-mediated adhesion of cells to the extracellular matrix, increase the tumor cell resistance to IR [129]. As a result, classical therapies become progressively ineffective toward these CSC-enriched tumors [130].

Few studies reported Wee1 inhibition in the particular case of CSCs inducing radio-sensitization in glioblastoma [67,131]. However, these findings support the concept that the overexpression of checkpoint inhibitors, such as Wee1, could be an adopted protection mechanism in CSCs with sublethal damage induced by conventional radio- and chemotherapy. Therefore, targeting the DNA damage checkpoint response in CSC may sensitize these cells to DNA-damaging techniques and overcome tumor resistances [132].

## 7. Wee1 Inhibitors

Wee1 represents an optimal target for the inhibition of the G2-M checkpoint in order to potentiate both radio- and chemotherapy.

In fact, although the purpose of the majority of anti-cancer therapeutic strategies involves cell cycle arrest, Wee1 kinase inhibition triggers mitosis and induces genomic instability, driving cells to follow a replication cycle, with consequential apoptosis for mitotic catastrophe.

This strategy may allow for the dose reduction of conventional therapies, leading to a reduction in toxic side effects while maintaining clinical efficacy; moreover, it may sensitize tumors with poor prognosis to conventional therapies.

Recent studies demonstrated that Wee1 inhibition can be reached with low cytotoxicity through rational drug design [133,134]. The only Wee1 inhibitor that is currently used in clinical trials (query on ClinicalTrials.gov, July 2021) (Accessed on 22 September 2021) is AZD1775, combined with DNA damage agents or radiotherapy, which is tolerable and demonstrates promising anticancer activity.

In addition to AZD1775, hundreds of compounds were reported to have inhibitory activity against Wee1 kinase. A recent study classified the Wee1 inhibitors that are reported in scientific literature into five groups on the basis of their chemical core structure (pyridopyrimidine derivatives, pyrazolopyrimidinone derivatives, pyrrolocarbazole derivatives, pyrimidine-based tricyclic molecules and vanillates) [135].

The first small molecule to be reported bearing the pyridopyrimidine core was PD0166285 [87] Although it showed a potent Wee1 inhibition activity (IC50 = 24 nM) in various cancer cell lines and xenografts [67,87,136,137,138], its clinical application is limited because of its non-selective Wee1 inhibition. In fact, PD0166285 presents a broad spectrum of inhibitory activity on several tyrosine kinases, including CHK1, MYT1, c-Src, EGFR, FGFR1 and PDGFR [87,135]. Due to its poor selectivity, PD0166285’s clinical application is limited. Furthermore, starting from the PD0166285 structure, several pyridopyrimidine derivates were synthesized while trying to increase the selectivity for Wee1, but most of them were found to preserve a potent inhibitory activity against c-Src [87,139,140,141].

Among the Wee1 inhibitors that have the pyrazolopyrimidinone scaffold, there is the known AZD1775. A series of pyrazolopyrimidinone analogs were synthetized [142] and, in 2016, Matheson and collaborators reported a compound (CJM-061) that showed the same Wee1 inhibitory efficacy of AZD1775, but had reduced single-agent cytotoxicity in medulloblastoma cells [133]. On this basis, Matheson et al. developed a series of potent pyrazolopyrimidinone-based Wee1 inhibitors; in particular, they found a compound that showed a stronger inhibition activity and reduced cytotoxicity compared to AZD1775 [134]. However, AZD1775 is the only Wee1 inhibitor that is currently used in clinical trials (query on ClinicalTrials.gov, July 2021).

The third class of Wee1 inhibitors is composed of the pyrrolocarbazole derivatives, all of which are analogs of the lead compound 4-phenylpyrrolecarbazole PD0407824, which is a powerful Wee1/Chk1 inhibitor. PD0407824 was a less potent Wee1 inhibitor compared to PD0166285 but was more selective for Wee1 and CHK1 than for c-Src [143]. Furthermore, it has only been tested on ovarian cancer, where it was shown to positively modulate the cis-platinum response [144]. On the basis of several structure–activity relationship studies [143,145,146], the analogs were designed in order to be more selective for Wee1 than for CHK1 [143]. Furthermore, the study of this class of Wee1 inhibitors has not been advanced into in vivo study, probably because of the broad spectrum of kinase inhibitor activity.

In 2014, Tong et al., aided by molecular modeling and structure–activity relationship studies, designed pyrimidine-based tricyclic molecules that showed potent Wee1 kinase inhibitor activity in both functional and mechanism-based cellular studies. The lead molecule, namely, 31, showed oral efficacy in the NCI-H1299 mouse xenograft model, potentiating the antiproliferative activity of irinotecan [147].

It is known that natural polyphenols, including polyphenols present in green tea (epigallocatechin gallate (EGCG)) [148], genistein [149] and curcumin [150], possess potential anticancer activity through inhibiting the proliferation, invasion and metastasis of tumoral cells and the induction of apoptosis, acting on different signaling pathways [148,149,150,151,152,153,154,155].

Starting from the core structure of polyphenols, Lamoral-Theys’ group designed and synthesized several di- and trivanillate compounds, with some of them presenting WEE1 inhibitory and anti-tumor activities, probably due to their inhibitory activity against the Aurora A/B/C responsible [156].

Table 2 summerizes the above reported evidence.

## 8. Wee1 Inhibition Sensitizes Response to Chemo/Radiotherapy

Among the Wee1 inhibitors, only AZD1775 is currently used in clinical trials. Several studies show its ability to potentiate the activity of chemotherapeutic drugs and radiotherapy. Several studies indicate that AZD1775’s sensitizing effect is selective only in p53-deficient tumors [142,157,158], although other evidence demonstrates that Wee1 inhibition can independently sensitize cancer cells to chemotherapeutics via p53 functionality [91].

In particular, in advanced squamous cell carcinoma of the head and neck (HNSCC), it was shown that a combination of AZD1775 and cisplatin can overcome cisplatin resistance, which is particularly high for patients whose tumor presents a mutation in the TP53 gene [159]. Other studies suggest a similar sensitizing effect in the radiation response in pontine gliomas [160], glioblastoma [131] and pancreatic cancer [161]. So far, there are 11 clinical trials testing AZD1775 in combination with various chemotherapeutic agents, 5 in combination with both chemo and radiotherapy and 2 in combination with only radiotherapy. Table 3 lists only the completed clinical trials with reported results.

## 9. Conclusions

Developing novel strategies to contrast local tumor cell survival and to, consequently, avoid the insurgence of metastasis is the principal aim of the researchers operating in the oncology field. As functionally impaired p53 is one of the principal hallmarks of tumor cells, they rely on remaining checkpoints to survive and proliferate. From this perspective, Wee1 is surely a prominent target to use in clinical practice for its dual role as gatekeeper in both the S-phase and G2/M transition. While most kinase inhibition strategies aim to arrest the cell cycle to block proliferation, WEE1 inhibition allows for mitosis to occur in cells with heavy DNA damage. This phenomenon increases the amplification of genomic instability through cellular replication cycles, which makes replication errors permanent. Cell cycle progression, when DNA damage occurs, leads to cells quickly accumulating a great number of mutations that make them unable to survive. Thus, through its inhibition, it might be possible to increase the effectiveness of DNA-damaging treatments, such as radiotherapy, forcing tumor cells to enter into mitosis, even with damaged DNA. This, finally, leads them to mitotic catastrophe and subsequent cell death.

Wee1 inhibition may allow for the reduction of the dose of cytotoxic chemotherapy, thus improving the safety profiles. It may also be employed to sensitize resistant tumors to conventional therapies when they are not effective as monotherapy.

## Figures and Tables

**Table 1:** Bibliographic evidence about Wee1 expression in different cancer types.

WEE1 Expression	Other Molecular Modulation	Cancer Type	Clinical Significance	Methods	Ref.
↓ Wee1		Stromal breast cancer (phyllodes tumor (PT)) human tissue samples	Wee1 reduction suggests a potential role of Wee1 as a tumor suppressor	Immunohistochemistry	[57]
↓ Wee1		Colon carcinoma cell lines and human tissue samples	Wee1 suppression suggests a potential role of Wee1 in tumorigenesis	cDNA array, Northern blotting and semi-quantitative reverse transcription-PCR (RT-PCR)	[58]
↓ Wee1	↑ Cyclin B1/cdc2 complex	NSCLC human tissue samples	Wee1 reduction is associated with a poorer prognosis and a higher recurrence rate	Immunohistochemistry	[59]
↓ Wee1	↑ miR-195	Metastatic melanoma cell lines and human tissue samples	Wee1 expression in malignant melanoma is directly regulated by miR-195	Immunoblotting, quantitative real-time PCR and immunohistochemistry	[61]
↑ Wee1	↓ miR-101-3p	Hepatocellular carcinoma (HCC) cell lines and human tissue samples	Downregulation of Wee1 enhances the radiosensitivity of HCC cells	Quantitative real-time PCR, Western blotting and flowcytometry	[60]
↑ Wee1		Leukemia cell lines	Wee1 kinase inhibition by siRNA silencing or by specific inhibitors potently sensitizes myeloid and lymphoid leukemia cells to Ara-C	High-throughput siRNA screen and Western blotting	[65]
↑ Wee1		Luminal breast cancer cell lines	Wee1 kinase inhibition by specific inhibitors has therapeutic potentials	High-throughput siRNA screen, Western blotting and immunohistochemistry	[66]
↑ Wee1		Nasopharyngeal carcinoma (NPC) cell lines	Wee1 kinase inhibition by specific inhibitors has therapeutic potentials	Western blotting	[75]
↑ Wee1		Glioblastoma cell lines and human tissue samples	Wee1 kinase inhibition by siRNA or specific inhibitors causes cell death and sensitizes glioblastoma to ionizing radiation in vivo, suggesting it has a potential therapeutic target	In silico analysis of microarray data, immunofluorescence staining, immunohistochemistry and Western blotting	[67]
↑ Wee1 kinase activity	↑ Cyclin D1	Hepatocellular carcinoma (HCC) human tissue samples	Activation of cyclin D1, Cdk4, cyclin E, cyclin A and Wee1 may play important roles in the process of malignant transformation of cirrhosis to HCC	Evaluation of WEE1 kinase activity using autoradiography	[68]
↑ Wee1		Medulloblastoma cell lines and human tissue samples	Wee1 kinase inhibition by siRNA or specific inhibitors (MK-1775) potently inhibits tumor growth in vivo and sensitizes medulloblastoma cells to cisplatin in vitro, suggesting Wee1 as a potential therapeutic target	Gene expression analysis, high-throughput siRNA screen and Western blotting	[69]
↑ Wee1		Glioma human tissue samples	Wee1 expression is directly correlated with the malignancy grade in all types of gliomas, but it is inversely associated with prognosis in GBM	Immunohistochemistry	[71]
↑ Wee1		Pediatric high-grade glioma	Wee1 expression positively correlates with the glioma grade; the Wee1 inhibitor MK-1775 increases the radiation cytotoxic effect and prolongs survival for mice with engrafted, orthotopic glioma	Gene expression analysis and immunohistochemistry	[70]
↑ Wee1		Melanoma cell lines and human tissue samples	High expression of WEE1 is associated with poor prognosis and Wee1 silencing increases tumor cell death; Wee1 represents a potential therapeutic target in melanoma	Immunohistochemistry and Western blotting	[74]
↑ Wee1		Vulvar squamous cell carcinomas human tissue samples	High Wee1 expression is associated with poor histological differentiation and lymph node metastases	Immunohistochemistry and Wee1 silencing using siRNA	[73]
↑ Wee1		Acute myeloid leukemia (AML) cell lines and AML primary cells	Wee1 inhibition sensitizes AML cells to cytarabine in vitro; Wee1 represents a potential therapeutic target in AML	Integrated genomic analyses	[90]
↑ Wee1		Osteosarcoma cell lines, human osteoblasts and tumor samples	Wee1 inhibition sensitizing OS cells to irradiation-induced cell death	Gene-expression data analysis, immunohistochemistry and Western blotting	[86]
↑ Wee1		Multiple myeloma (MM) tissue samples and cell lines	Wee1 inhibition in combination with Bortezomib induces cell death in all cell lines more efficiently compared to the single agents	Quantitative real-time PCR (qPCR)	[89]
↑ Wee1		Ovarian carcinoma (OC) peritoneal effusion samples and cell lines	Wee1 is overexpressed in post-chemotherapy disease, suggesting a role in mediating disease progression and as a prognostic marker of poor survival; Wee1 is a potential therapeutic target in OC	Immunohistochemistry and Western blotting	[72]

**Table 2:** Wee1 inhibitors. Standard color coding for atoms is used (black—carbon, red—oxygen, blue—nitrogen, green—chlorine, brown—fluorine).

	Core Chemical Structure	Lead Compounds	CHK1 IC_50_ (nM)	WEE1 IC_50_ (nM)	References
Pyridopyrimidine derivatives	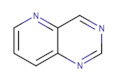	PD0166285 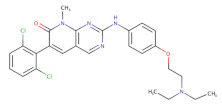	72	24	[86]
Pyrazolopyrimidinone derivatives	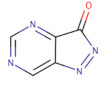	CJM-061 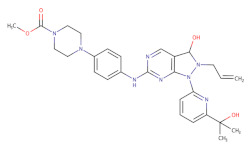	-	2.8	[133]
		AZD1775 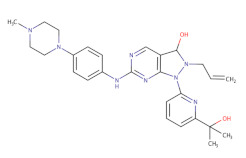	-	1.9	[133]
Pyrrolocarbazole derivatives	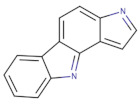	PD0407824 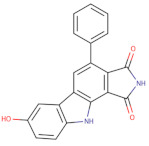	47	97	[143]
Pyrimidine-based tricyclic molecules	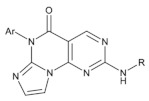	31 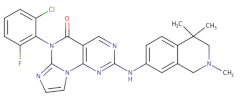	-	H1299 EC = 230	[147]
Vanillates	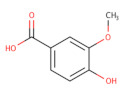	32 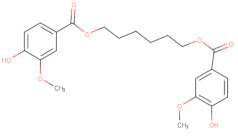	-	20,000	[156]
		33 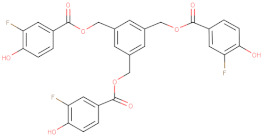	-	7120	[156]

**Table 3:** Registered clinical trial on Wee1 inhibitors.

	Title	Conditions	Interventions	ClinicalTrial.govIdentifier Number
1	WEE1 Inhibitor with Cisplatin and Radiotherapy: ATrial in Head and Neck Cancer	- Hypopharynx squamous cell carcinoma - Oral cavity squamous cell carcinoma - Larynx cancer	- AZD1775 - Cisplatin - Radiotherapy	NCT03028766
2	WEE1 Inhibitor AZD1775 with or without Cytarabine in Treating Patients with Advanced Acute Myeloid Leukemia or Myelodysplastic Syndrome	- Chronic myelomonocytic leukemia - Myelodysplastic syndrome with isolated del(5q) - Myelodysplastic/myeloproliferative neoplasm - Previously treated myelodysplastic syndrome - Recurrent adult acute myeloid leukemia - Untreated adult acute myeloid leukemia	- AZD1775 - Cytarabine	NCT02666950
3	Phase Ib Study AZD1775 in Combination with Carboplatin and Paclitaxel in Adult Asian Patients with Solid Tumours	Advanced solid tumors	- AZD1775 - Paclitaxel - Carboplatin	NCT02341456
4	Cisplatin with or without WEE1 Inhibitor MK-1775 in Treating Patients with Recurrent or Metastatic Head and Neck Cancer	- Recurrent hypopharyngeal squamous cell carcinoma - Recurrent laryngeal squamous cell carcinoma - Recurrent laryngeal verrucous carcinoma - Recurrent lip and oral cavity squamous cell carcinoma - Recurrent metastatic squamous cell carcinoma in the neck with an occult primary - another 24 tumors	- MK-1775 - Cisplatin	NCT02196168
5	Ph II Trial of Carboplatin and Pemetrexed with or without AZD1775 for Untreated Lung Cancer	Previously untreated stage IV non-squamous non-small cell lung cancer	- AZD1775 - Pemetrexed - Carboplatin	NCT02087241
6	Dose Escalation Trial of AZD1775 and Gemcitabine (+Radiation) for Unresectable Adenocarcinoma of the Pancreas	Adenocarcinoma of the pancreas	- AZD-1775 - Gemcitabine - Radiotherapy	NCT02037230
7	A Study of MK-1775 in Combination with Topotecan/Cisplatin in Participants with Cervical Cancer (MK-1775-008)	Cervical cancer	- MK-1775 - Topotecan - Cisplatin	NCT01076400
8	A Dose Escalation Study of MK-1775 in Combination with Either Gemcitabine, Cisplatin, or Carboplatin in Adults with Advanced Solid Tumors (MK-1775-001)	Solid tumors	- MK-1775 - Gemcitabine - Cisplatin - Carboplatin	NCT00648648

## Data Availability

Data supporting reported evidences can be obtained from the original sources (please see reference), data from clinical trials can be retrieved from www.ClinicalTrial.gov.
